# Shared Decision-Making: The More the Patient Knows, the Better the Decision that Is Made

**DOI:** 10.31083/j.rcm2408232

**Published:** 2023-08-11

**Authors:** Lloyd W Klein

**Affiliations:** ^1^Department of Medicine, Cardiology Division, University of California, San Francisco, San Francisco, CA 94143, USA

Shared decision-making (SDM) is a strategic means of reaching consensus with patients to incorporate their health preferences and goals into their treatment. By supporting patient autonomy and engagement through emphasizing a patient-centered approach, the patient and family become active participants in their healthcare, leading to improved outcomes and satisfaction. The process is intended to bring into focus individual patient goals within the context of the evidence base. The potential benefits and harms of alternative approaches are articulated and the patient’s values and preferences considered [1, 2].

The critical condition of SDM is that the clinician and patient engage in a dialogue to jointly develop a plan, with reciprocated sharing of information and patient empowerment. “Sharing” means bi-directional transfer of knowledge, particularly involving patient preference and a process of deliberation. Respect for patients’ goals and preferences and using them to guide treatments when compatible with professional guidelines and the evidence base is the hallmark of successful SDM. Effective SDM requires that patients possess sufficiently accurate information to ask informed questions and express personal values [1, 2, 3, 4].

How and when these goals are operationalized are critical attributes of the process. SDM is optimally employed in situations in which there is more than one valid treatment option, sometimes with a complicated favorable/unfavorable balance, when the choice between options involves the patient weighing the trade-offs according to their personal inclinations and values. In the presence of scientific uncertainty, patient preference legitimately takes on greater importance [1, 5]. To assure that the information provided to a patient is understandable and balanced, patient decision aids (PDAs) have been developed that foster patient comprehension of the available choices [6]. SDM affords many benefits to the patient-physician relationship, and enhances communication and satisfaction [7, 8].

In this issue of Reviews in Cardiovascular Medicine, Zheng *et al*. [9] present a meta-analysis of 10 studies showing that SDM utilizing PDAs improved the knowledge, decision satisfaction, participation and medical outcomes of patients, and reduced decision-making conflict. The PDAs format, and to some extent content, varied from study to study. All of the studies concluded that PDAs improved patient knowledge and most showed improvement in decision conflict and patient satisfaction. These endpoints were accompanied by better patient acceptance, positive attitude, and quality of life.

These are important conclusions because many skeptics claim that there is nothing shared about SDM but rather the communication of bias. Their argument is that no matter how sincere the physician may be in presenting a balanced approach to treatment options, it is an inherently unequal relationship. This meta-analysis shows that when the process is carried out in a way that respects patient choice, everyone benefits. Physician reluctance to acquiesce to an option they don’t consider the “best” one is the most prominent obstacle to SDM. Naturally, proficient clinicians know the clinical evidence better than a patient ever can and have anecdotal experience that goes beyond clinical trials. Learning more about their disease in a structured learning situation depends on a substantial level of patient cognition and unbiased education. PDAs are intended to assure that balanced information is presented, and some studies use scales to evaluate the SDM process objectively [10, 11]. Giving patient access to unbiased information rather than controlling what the patient knows reflects a commitment to sharing; but exactly how the information influences patient treatment choice is largely unknown. Well-designed decision aids that present patients with all contemporary therapeutic options may be useful adjuncts to practice in many complex medical situations [12]. The presentation of balanced options with a PDA might suggest that medical science has not fully determined what the best treatment option is [13, 14, 15], a recognition that can be upsetting to patients, who may seek a second opinion from a more confident, if less patient-oriented, medical professional.

It’s difficult to admit to the patient or the referring primary “I’m not sure” in an environment which values, and even rewards, the appearance of certainty. SDM fosters discussion that allows a shift in thinking from a need to exhibit certainty to a goal of accurately portraying what is known and what isn’t, and giving the patient a say in their own lives. SDM assumes that patients are well-informed about their health condition, treatment options, and potential risks and benefits. However, patients may have varying levels of health literacy or may not be sufficiently prepared to participate actively in the decision-making process. In such cases, it may be challenging to achieve true SDM. Shared decision making may not be equally accessible to all patients. Factors such as education level, socioeconomic status, language barriers, and cultural differences can influence the extent to which patients can effectively participate in the decision-making process. Inequities in access to information can contribute to healthcare disparities.

Expressing their concerns to an authority figure may be daunting to many patients. Some cardiologists are unwilling or unable to explain their rationale, while others may intentionally use the power differential to influence the patient into the strategy they favor. An example of a cynical application of SDM is when the cardiologist asserts that a stent was placed “because the patient wanted it”, knowing full well nothing was done to inform them of alternatives, or on occasion, after misleading information was provided or not corrected.

Risk avoidance is a powerful determinant of decisions; objective decisions about oneself can be difficult for anyone when the riskier choice also leads to better results overall. Selecting a treatment option based on clinical results may conflict with the emotional concerns that are raised by a consideration of risk. What patient can dispassionately judge the balance between an increased risk of stroke versus improved survival, for example? But if the concept is misapplied to merely “convince” a patient of a certain strategy they are hesitant to take, even if intended with their best interest, is that ethical? And, is it truly “shared”? There’s always an element of randomness to medical outcomes that can’t be controlled. Even well educated patients may be uncomfortable with dealing with the uncertainties inherent in medical decisions. No clinician can deny (to oneself or to the patient) that the best-informed decision doesn’t always yield the best outcome [16]; leaving the choice to the patient therefore cannot be merely a tactic to assuage the physician’s conscience. When patients are actively involved in decision-making, they may experience decisional conflict or uncertainty. Making complex medical decisions can be overwhelming, and patients may struggle with weighing the pros and cons of different treatment options. The responsibility of decision-making can sometimes lead to anxiety or distress.

There is increasingly an unnamed third participant in medical decision-making: the health system that evaluates quality of care. Quality assurance programs aren’t forgiving when the guideline recommended strategy isn’t followed, considering an absence of compliance to be due to the physician’s lack of competence, not the desire to work with patient desires. Certainly, when the chosen strategy is less aggressive, and hence less costly than the recommendation, that divergence is often overlooked. But what if it costs more? Suppose the patient requests a revascularization procedure in a situation with uncertain evidence to support it? In some employment models, physicians whose patients tend to receive the more expensive treatment options may not be renewed. The resulting weaponization of quality and its corollaries, diminishing variations in management and cost, are fabricated directly in opposition to SDM. The fee-for-service system has been constructed to produce revenue and limit costs, not necessarily to be responsive to what people desire.

It is important for healthcare providers to be aware of these drawbacks and work towards mitigating them to ensure effective and patient-centered care. In today’s complex medical environment, doing what is best for our patients isn’t as easy as it sounds. Presenting unbiased information is the key to the successful physician-patient relationship, and “keeping one’s thumb off the scale” a crucial component. A 1970s clothing retailer used television commercials with the tagline: “an educated consumer is our best customer”, meaning that the more the consumer knew, the more they could see for themselves who provided the best quality and value. I have always believed in that slogan and practice accordingly; but the details of how the patient and family are informed make all the difference, and require our vigilance to assure an authentic choice.
